# Knowledge, attitude and practice towards sexually transmitted diseases amongst the inmates of women shelters homes at Klang Valley

**DOI:** 10.1186/s12889-019-6863-5

**Published:** 2019-06-13

**Authors:** Noraziah Mohamad Zin, Ismarulyusda Ishak, Kasturi Manoharan

**Affiliations:** 1 Centre of Healthy Aging and Wellness, Universiti Kebangsaan Malaysia, Jalan Raja Muda Abdul Aziz, 50300 Kuala Lumpur, Malaysia; 2 Centre for Health and Applied Sciences, Universiti Kebangsaan Malaysia, Jalan Raja Muda Abdul Aziz, 50300 Kuala Lumpur, Malaysia

**Keywords:** Knowledge, Attitude, Practice, Sexually transmitted diseases, Women shelter home

## Abstract

**Background:**

Previous studies show that there is a changing trend of sexual and reproductive behaviour among youth and this requires more attention and awareness especially on sexually transmitted diseases (STD). This study was carried out to evaluate the knowledge, attitude and practice of sexually transmitted diseases among selected inmates of women shelter homes.

**Methods:**

A cross-sectional study was carried out by involving 60 participants whom aged in between 13 to 25 years old. The questionnaires were developed in ‘Bahasa Melayu’ and it has been anonymous guided questionnaires.

**Results:**

The result showed that the mean age of the participants was 17.9 years old and most of the participants have completed secondary school (91.7%). Overall, the level of knowledge of participants on STDs were classified into three groups; ‘high knowledge’ (33.3%), ‘medium knowledge’ (35.0%) and ‘low knowledge’ (31.7%). The majority have heard of HIV/AIDS (95%) but with respect to other STDs was less well known. Whereas, the mean score for attitude was 23.1 out total 25. Their knowledge level was not influenced by their age (*p* = 0.61) and socio-economic status (*p* = 0.85). However, their attitude was influenced by their age (*p* < 0.05).

**Conclusion:**

Knowledge on non-HIV STDs is still lacking and risky behaviours have been practiced. Although there were high level of knowledge and attitude among them but their practice on sexuality contradicts it especially on contraceptive use and pre-marital sex.

## Background

Sexually transmitted diseases (STDs) of youths has emerged as an issue of great concern particularly to the policy-makers, health service providers, family, community and the individual him or herself. Malaysia youth is defined as people from 15 to 40 years old and this phase is considered as a transitional phase where the individuals will experience rapid physical, mental and social development [1]. Developing countries undergo a great burden because of the prevalence of non-curable viral from sexually transmitted diseases (STD), increased travel and behavioural change. There is a changing trend of sexual and reproductive behaviour among youth and this requires more attention and awareness from the health care professionals to provide necessary sexual or reproductive care and education [2]. STD are diseases caused by either bacteria, virus or parasites which spread predominantly by sexual contact including vaginal, anal and oral sex. STD also can spread through non-sexual means such as via blood, blood product or from mother to child during pregnancy and childbirth [3]. According to UNICEF, the average phase where many people get their first experience of sexually activity is during their youth life [4]. For instance, the National Health and Morbidity Survey (Malaysia), showed that 7.3% of the respondents whom were 13–17 years old already had sex experiences [5]. Unfortunately, during this phase many studies have proved that the youth are poorly informed about STD and its prevention [6–8]. According to Women Aid Organisation, women shelter home is a place which provide temporary shelter, physical and emotional support for the affected women. It could be a victim of rape, domestic violence or women with sexual discipline problem. These shelter homes provide the necessary emotional, mental and social support services that empower and enable them to live a better life in future [9]. Hence, this research was carried out to study the knowledge, attitude and practice towards sexually transmitted diseases amongst the inmates of women shelter homes at Klang Valley. Many Muslim youths were sent to the women shelter homes by their family members due to premarital sexual relationship that led to premarital pregnancies. Besides that, comparison of knowledge, attitude and practice of sexually transmitted diseases to age and socioeconomic status were also done. In addition, this study also wanted to create awareness to emphasis on other types of STD apart from HIV/AIDS.

## Methods

### Study design

A cross-sectional study was conducted by using self-administered questionnaires. The researcher gave guidance by reading out the questions to the participants for better understanding, but the answers solely depends on the participants as it was self-administered questionnaire. The respondent’s identity remains anonymous throughout the whole study and has obtained approval from Research Ethics, Universiti Kebangsaan Malaysia (UKM): UKM PPI/111/8/JEP-2017-655.

### Study population and sampling

Inmates of women shelter homes were the target group. By simple random sampling, two shelter homes were assigned. One from Kuang town (Gombak district) and another from Shah Alam city (Petaling distict) were selected among the seven-shortlisted women shelter homes in Klang Valley. These seven shelter homes were shortlisted upon the approval from management to conduct the study. A written informed consent was taken prior to answering the questionnaire. In addition, an informed consent for legal guardian was given for participants below 18 years old. All the inmates who were present during data collections time from these two shelter homes participated in this study. A few inmates were away to hospital for routine check-up and were exluded in this study.

### Questionnaire development

This questionnaire was developed in ‘Bahasa Melayu’ (national language of Malaysia) and consist three parts (Part A, Part B and Part C). The questions in this questionnaire was built upon existing literature by exploring respondent’s knowledge, attitude and practice about STD [8]. Pilot study was carried out to pre-test this questionnaire. Part A is the demographic section with 7 questions. Whereas Part B have 13 questions which play its role to test the respondent’s knowledge on sexuality and sexually transmitted diseases (Question 1–9). Meanwhile, Question 10–13 from Part B, indicating the respondent’s sexual practice. Finally, Part C have 5 questions which was helpful to know the respondent’s attitude towards sexuality, premarital sexual activity, multiple sex partner and sexually transmitted diseases. The response option for questions in this part was in categories such as ‘strongly disagree / disagree/ neutral/ agree/ strongly agree’, (α = 0.71).

### Data analysis

The data was analysed by using Statistical Package for the Social Science (SPSS). Descriptive analysis was carried out for the demographic variables then followed by comparisons among groups was done by using One-way analysis of variance (ANOVA) test. Hence, one-way ANOVA was employed to compare the level of knowledge on STD according to age group and total family income. Based on consensus from the expert, the level of participant’s knowledge on sexually transmitted diseases were classified into three groups. Furthermore, those who scored below 9 was considered having ‘low knowledge’, above 13 classified as ‘high knowledge’ and the scores in between was considered as ‘medium knowledge’. Besides that, correlation test and Chi Square was employed to determine association between knowledge, attitude and practice. Statistical significance of 0.05 was used.

## Result

A total of 60 participants were involved which divided into several criteria (age, origin, occupation, religion, marital status, education level, family income per month) and showed in demographic data (Table 1). All participants were Muslim, not working and single. The mean age for the participants in this study was 17.9 years old with the youngest of 13 years old and eldest was 25 years old. For the state of origin, Selangor (26.7%; *n* = 16) has the highest participants in this study meanwhile Johor, Kelantan, Kedah and Terengganu recorded the least (6.2%; *n* = 4). All the participants were single, Islam and not working. Most of them completed secondary school (91.7%; *n* = 55) and the highly reported total family income was ranged in between RM1001-RM3000 (36.7%; *n* = 22).

**Table 1:** General demographic and knowledge level of the participants

Demographic	Characteristic			Knowledge Level			
	n = 60	%	Mean	SD	df	F	*p*
Age (years)							
Dec-16	21	35	10.9	2.34			
17–21	33	55	11	2.85	2	0.49	0.61
22–26	6	10	9.83	2.48			
Origin							
Selangor	16	26.7	12.0	2.88			
WPKL	6	10	9.6	3.14			
Negeri Sembilan	8	13.3	11.1	2.23			
Johor	4	6.7	10.0	3.36	8	1.24	0.29
Kelantan	4	6.7	8.2	2.36			
Terengganu	4	6.7	10.7	0.95			
Perak	5	8.3	10.8	2.38			
Pahang	9	15	11.2	2.27			
Kedah	4	6.7	10.0	2.16			
Education Level							
Primary	1	1.7	6.0	–			
Secondary	55	91.7	10.8	2.59	3	2.11	0.11
STPM	3	5	11.33	1.15			
Degree	1	1.7	15.00	–			
Total Family Income							
RM500-RM1000	11	18.3	10.36	2.38			
RM1001-RM3000	22	36.7	11.19	2.79	3	0.26	0.85
RM3001-RM5000	18	30	10.58	2.99			
> RM5000	9	15	11.22	1.86			

### Knowledge level of sexually transmitted disease

Overall, the level of knowledge of participants on sexually transmitted diseases were classified into three groups; ‘high knowledge’ (33.3%), ‘medium knowledge’ (35.0%) and ‘low knowledge’ (31.7%) as showed in (Table 2). The mean score of knowledge was 10.9. The lowest score was 5 and highest was 15 out of total 16 scores. When comes to the knowledge on types of sexually transmitted diseases (Table 3), the highest identified STD was AIDS which is 95% (*n* = 57) whereas only 3.3% (*n* = 2) managed to identify Trichomoniasis as STD. Meanwhile, the other STD were identified as following; Syphilis (66.7%; *n* = 40), Gonorrhoea (43.3%; *n* = 26), Genital Herpes (30.0%; *n* = 18) and Chlamydia (26.7%; *n* = 16). In contrast, some even classified Dengue Fever (5%; *n* = 3), Urethritis (13.3%; *n* = 8), Scabies (5%; n = 3), Brucellosis (1.7%; *n* = 1) as STD. Apart from types of STD, the participants also were tested for their knowledge on the risk and side effect of STD. About 91.7% (*n* = 55) stated that STD can cause infertility to both men and women whereas only 8.3% (n = 5) said that only women affected by infertility root from STD. Meanwhile, about 50% (*n* = 30) said STD can cause cancer but almost another half (41.7%, *n* = 25) were not sure about it. When it comes to contraceptive, 55% (*n* = 33) believed that it can prevent STD and 6.7% (*n* = 4) denied it. In regards with the curability of STD, 80% (*n* = 48) believed it cannot be cured. A high percentage of the participants, 95% (*n* = 57) agreed on the increased risk of STD due to having multiple sex partner. The transmission of STD from mother to baby during pregnancy or delivery were fairly known by the participants as well, 71.7% (*n* = 43). In addition, hospital and internet appear to be the most common source of information on sexuality and STD. The ANOVA was statistically not significant for both, indicating that the knowledge level of STD is not significantly different by the age of participants, *F* (2,57) = 0.49, *p* = 0.61 and total family income STD, *F* (3,56) = 0.26, *p* = 0.85 which is shown in (Table 1).

**Table 2:** Knowledge level on sexually transmitted diseases

Level of Knowledge	Characteristic (*n* = 60)		
	Scores	n	%
Low	< 9	19	31.7
Medium	9 to 13	21	35.0
High	>13	20	33.3

**Table 3:** Knowledge on types of sexually transmitted diseases

Type of STDs	Characteristic	
	Yes *n* (%)	No *n* (%)
Gonorrhoea	26 (43.3)	34 (56.7)
Syphilis	40 (66.7)	20 (33.3)
HIV/AIDS	57 (95)	3 (5)
Dengue Fever	3 (5)	57 (95)
Trichomoniasis	2 (3.3)	58 (96.7)
Urethritis	8 (13.3)	52 (86.7)
Scabies	3 (5)	57 (95)
Brucellosis	1 (1.7)	59 (98.3)
Chlamydia	16 (26.7)	44 (73.3)
Genital Herpes	18 (30)	42 (70)

*Dengue Fever, Urethritis, Scabies and Brucellosis are not sexually transmitted diseases

### Attitude towards sexually transmitted disease

The mean score for attitude was 23.1 out total 25. A high percentage of the participants were opposing premarital sex (88.3%; *n* = 53) and multiple sex partner (90%; *n* = 54) respectively. Moreover, a good number of participants agreed with avoidance of sexually activity with STD infected person (93.3%; *n* = 55), seek of medical help for STD infected person (96.7%; *n* = 58) and acceptance to see doctor if there are any symptoms of STD (96.7%; *n* = 58) as per shown in Table 4. One-way ANOVA of attitude with age and total family income showed that there was statistically significant at *F* (2,57) = 5.82, *p* < 0.05. However, in (Table 5), the ANOVA was not statistically significant at *F* (3,56) = 0.37, *p* = 0.77 for the attitude of participants towards STD according to family income. Hence, attitude is not affected by their total family income.

**Table 4:** Attitude towards sexually transmitted diseases

Attitude	Strongly Agree	Agree	Neutral	Disagree	Strongly disagree
	% (n)	% (n)	% (n)	% (n)	% (n)
Avoiding ‘being together’ (sexual intercourse) with someone who has sexually transmitted diseases is necessary because it can be contagious	70.0 (42)	23.3 (14)	5.0 (3)	–	1.7 (1)
An STD infected person should seek medical help	75.0 (45)	21.7 (13)	1.7 (1)	1.7 (1)	–
What do you think about sex before marriage?	–	–	11.7 (7)	15 (9)	73.3 (44)
What do you think about multiple sexual partners (more than one)?	1.7 (1)	3.3 (2)	5.0 (3)	20.0 (12)	70.0 (42)
If you have symptoms of sexual illness, are you willing to see a doctor?	65.0 (39)	31.7 (19)	3.3 (2)	–	–

**Table 5:** Level of attitude with age and total family income

Characteristic	n	Mean	SD	df	F	p
Age Group						
12–16	21	23.33	1.56	2	5.82	0.005
17–21	33	23.55	1.80			
22–26	6	20.83	2.56			
Total Family Income						
RM500-RM1000	11	23.55	1.96	3	0.37	0.77
RM1001-RM3000	22	23.43	1.69			
RM3001-RM5000	18	22.79	2.07			
> RM5000	9	23.11	2.37			

### Practices of participants in sexual activity

Among the participants, 43% (*n* = 26) have three or more partners within these 3 years. When comes to the causes of participants engage in sexual activity varies, the most common reason was upon their own decision (*n* = 43), forced by their partner (*n* = 23) and the desire to try it (*n* = 23). Besides that, the participant’s practice of using contraceptive, about 77% (*n* = 46) admit that they don’t use any contraceptive.

### Association of knowledge, attitude & practice

A bivariate Pearson’s correlation was used to evaluate the possible association of knowledge and attitude. There was a positive but weak correlation between knowledge and attitude, *r* (58) = 0.36, *p* < 0.05. Apart from that, a chi-square test of contingencies was carried out to evaluate does the level of knowledge associate with the practice of participants having multiple sex partner. The chi-square test was statically not significant, χ ^2^ (6, *N* = 60) =15.84, *p* = 0.20. Thus, the level of knowledge of the participants does not influence their practice of having multiple sex partner.

## Discussion

According to Centers for Disease Control and Prevention (CDC), South and Southeast Asia have reported the largest number of new curable cases of STDs [10]. Thus, this shows that STDs in our region is on rise. However, the knowledge and awareness among public regarding this is still questionable especially for those who are at risk. The main purpose of conducting this study on women whom staying at shelter homes is due to their risky exposure to sexual act in past. Most of them involved in sexually activity without an appropriate knowledge about its negative side [11]. The mean age of participants in this study was 17.9 years old. However, by this age they have been already exposed to sexually activity. Thus, their first sexual debut should be younger than this. According to the National Health and Morbidity Survey (Malaysia), male (35%) and female (27%) already had sex before 14 years old [5]. While another report showed that 8.3% of school students have already had sex with the mean age of 15 years old for their first sexual encounter [12]. On a separate study, they found that the younger a person start their sexual activity, the higher their risk of having multiple sex partner [13]. To support this a similar finding was reported by the National Health and Morbidity Survey (Malaysia), 21% of male and 11% of female are having multiple sex partner [5]. In addition, on a study done in China showed that female who had first debut to sex at age younger than 18 years old are more likely to have multiple sex partner compared to those who started at 19 years old or older. Thereby, significantly increasing their risk for STDs and pregnancy [14].

### Knowledge

About one third of the participants (*n* = 19) scored ‘low knowledge’ in this study (Table 2) and they might be engaging in sexual activity without accurate information which will be resulting in risky practice and side effects such as STD and unwanted pregnancy [15]. A study showed that the teenagers are having high-risk pregnancies due to low education level and low socioeconomic status [16]. In contrast, this study shows that the level of knowledge regarding STD is not influenced by the age of participants or their socioeconomic status although previous finding said about the understanding regarding sexuality depend on the age and life status [17]. In this study, HIV/AIDS remains as the most famously identified STD followed by Syphilis, Gonorrhoea, Genital Herpes, Chlamydia and Trichomoniasis and this finding also supported by few previous studies in Malaysia [12, 17]. It has been well observed that HIV/AIDS always given more importance compared to other STDs during campaigns or health promotions. Surprisingly, about 5% participants from our study believed that, the dengue fever categorised as one of STDs, although it already documented mosquito as primary spreading vector but not by infected human with STDs [7]. This clearly shows that the unawareness and low knowledge level about STD in young people in certain places still occur although at low percentage. However, there was high percentage (95%) believe on having multiple sex partner increases the risk of STD and showed similar with other previous study [18]. A large proportion of this study have a good knowledge on transmission of STD from mother to child during pregnancy or childbirth (71.7%). In regards with source of information, hospital and internet was on leading and the least was family and radio. Surprisingly schools had very limited role in spreading the information. Current sexual education is not taught in depth and only cover certain aspects such as sexual organs, sexual intercourse and fertilization which results in limited guidance for youth to face the real world [19]. Since these participants were from shelter homes, some educational talks on health have been conducted by Ministry of Health. Thus, it may influence the high reporting rate of hospitals. Internet is the most common sources of sexual information but unfortunately parents who were supposed to be the main source of information remain as the least one [20]. In addition, Folasayo and co-workers also had similar findings with internet is the most common source of information compared to family members [7]. These facts emphasise parents should create a situation for an open parent-adolescent communication about sexually related matter.

### Attitude

Overall, the mean score for attitude in this study was 23.1 out of 25, which was relatively high. Approximately, 93% agreed about avoidance of sexually activity with STD infected person would prevent the spread of it. Even on a few more studies done at United States of America, showed high percentage which is 71.4% agreed to it [21, 22]. This study found that about 96.7% agreed that an STD infected person should seek medical help. In addition, same percentage of the participants also agreed that are willing to seek medical help if they have any STD symptoms. This shows that, they are open to receive treatment and seek medical help when needed. A previous study elsewhere in Malaysia showed similar findings of 85.5% agreed to seek immediate treatment if they or their partners have STD symptoms [7]. In regards with perception on premarital sex, 88.3% from this study opposed it. A previous study also showed a similar finding which recorded negative attitude towards premarital sex [23]. Culture and religion faith about premarital sex is unacceptable in Malaysia so their attitude in this matter is based on the surroundings. For Muslims, it is strongly forbidden to have sex before marriage and especially when two non-spouse have sex which is known as ‘zina’, one of the biggest sin. In contrast, most female undergraduate in China thought premarital sex is acceptable [14]. When comes to multiple sex partner, 90% opposed it but their practices are totally against it. Besides that, this study found that attitude of the participants was not influenced by their family socioeconomic status. In support to this, a study in Penang also found similar correlation [24]. Somehow, their attitude is influenced by their age. As they get older, with increase of knowledge and awareness, they show better attitudes.

### Practice

Despite of having high knowledge (95%) and attitude (90%) on the risk of having multiple sex partner, 55% of the participants admitted having more than one partner. Specifically, 43% participants had three or more than that as their sex partner. Other studies also showed those with moderate level of knowledge on having multiple sex partner also implemented their practice poorly. Therefore, having sufficient knowledge alone does not always guarantee a person’s behaviour and practice [25]. The top three factors that were associated with participant’s involvement in sexual activities are upon their own decision (mutual decision with partner), forced by partner and followed by desire to try. In many Asian culture, reproductive health decision making is based upon male authority. Studies have shown that women are with less power to refuse sex when forced by their partner [26]. This would be the reason many agreed when forced by their partner. Although 55% of the participants believed that contraceptive can prevent STD, but almost 77% admitted that they do not use any type of contraceptive during sexual act. A few more studies also corroborate this, a high tendency for non-condom sex at first sexual act and on most occasion of sexual intercourse [5, 27, 28]. Furthermore, a study in Brazil also reported the same, although 94% were aware of the use of condom in prevention of STD but only 34% practice using it during sexual intercourse [8]. Contraceptive will prevent STDs and arrest unintended pregnancies [29]. Unprotected sexual activity predisposes the adolescents to serious consequences later in life [30]. Apart from that, about 88.3% in this study showed their attitude of opposing premarital sex, but their practice was contrary because 100% of the participants in this study were single and had sexual experience before this.

### Limitation

The result obtained in this study only represent the small community (*n* = 60) in two selected shelter homes and not the real scenario in the whole community of youth women staying at shelter homes in Malaysia. Although it was a guided questionnaire, but it was self-filled up by the participants. Therefore, there might be under-reporting on some questions related to sexual behaviours since sex is a taboo topic in Malaysia. The possibility of recall bias was also present due to the close-ended questions and multiple choice of answers. There might be some assuming answers took place.

## Conclusion

This study described the knowledge, attitude and practice of sexually transmitted diseases among women at shelter homes. Based on our findings, although HIV/AIDS are most identified STD but other types of it remain obscure. Therefore, there is a need to educate public especially the youth regarding the other types of STDs apart from HIV/AIDS and as well as about contraceptive use. Besides that, a good collaboration between family, school and government agency should be created. Family and schools should be the main source of information regarding sexual health. Thus, Ministry of Education may consider review current sex education especially adding STDs in the syllabus. In addition, Ministry of Health also may consider improving health promotion about the other types of STDs as HIV/AIDS, equal amount of importance should be given. Besides that, a regular health screening on STDs and their treatments should be made more available. Adequate correct information is essential for the youth before they commence in sexual activity. This study also found that, although they have high level of knowledge and attitude, but their practice still contradicts. Hence, a future study may need to discover a better way to improve their practice level or to identify the barriers for them to practice their knowledge and attitude. Since all of the correspondence are Muslim and 88.3% of them are opposed to premarital sex, the status of their religion faith should be strengthened, monitored and evaluated. Furthermore, this is a cross-sectional study and the results can be descriptive only. Hence, exploratory study should be carried in future for better understanding on knowledge, attitude and practice of STD among these women.

## Abbreviations

AIDS: Acquired immunodeficiency syndrome
CDC: Centers for Disease Control and Prevention
HIV: Human immunodeficiency virus
STD: Sexual transmitted disease
χ ^2^: Chi-square
